# Automatic mandibular canal detection using a deep convolutional neural network

**DOI:** 10.1038/s41598-020-62586-8

**Published:** 2020-03-31

**Authors:** Gloria Hyunjung Kwak, Eun-Jung Kwak, Jae Min Song, Hae Ryoun Park, Yun-Hoa Jung, Bong-Hae Cho, Pan Hui, Jae Joon Hwang

**Affiliations:** 1 Department of Computer Science and Engineering, The Hong Kong University of Science and Technology, Pokfulam, Hong Kong; 2 National Dental Care Center for Persons with Special Needs, Seoul National University Dental Hospital, Seoul, Korea; 3 Department of oral and maxillofacial surgery, school of dentistry, Pusan National University, Pusan, Korea; 4 Department of Oral Pathology & BK21 PLUS Project, School of Dentistry, Pusan National University, Yangsan, Korea; 5 Department of Oral and Maxillofacial Radiology, School of Dentistry, Pusan National University, Dental and Life Science Institute, Yangsan, Korea; 6 Department of Computer Science, The University of Helsinki, Turku, Finland

**Keywords:** Dental radiology, Cone-beam computed tomography, Three-dimensional imaging

## Abstract

The practicability of deep learning techniques has been demonstrated by their successful implementation in varied fields, including diagnostic imaging for clinicians. In accordance with the increasing demands in the healthcare industry, techniques for automatic prediction and detection are being widely researched. Particularly in dentistry, for various reasons, automated mandibular canal detection has become highly desirable. The positioning of the inferior alveolar nerve (IAN), which is one of the major structures in the mandible, is crucial to prevent nerve injury during surgical procedures. However, automatic segmentation using Cone beam computed tomography (CBCT) poses certain difficulties, such as the complex appearance of the human skull, limited number of datasets, unclear edges, and noisy images. Using work-in-progress automation software, experiments were conducted with models based on 2D SegNet, 2D and 3D U-Nets as preliminary research for a dental segmentation automation tool. The 2D U-Net with adjacent images demonstrates higher global accuracy of 0.82 than naïve U-Net variants. The 2D SegNet showed the second highest global accuracy of 0.96, and the 3D U-Net showed the best global accuracy of 0.99. The automated canal detection system through deep learning will contribute significantly to efficient treatment planning and to reducing patients’ discomfort by a dentist. This study will be a preliminary report and an opportunity to explore the application of deep learning to other dental fields.

## Introduction

The inferior alveolar nerve (IAN), the third branch of the trigeminal nerve, is one of the major structures in the mandible that supplies sensation to the lower teeth. Moreover, it forms the mental nerve after passing through the mental foramen and supplies sensation to the chin and lower lip^1^. Finding the position of the IAN is a crucial step in implant installation, third molar extraction, and various other craniofacial surgeries including orthognathic surgery. Any injury to the IAN could result in temporary or permanent damage, where patients experience numbness and discomfort^2–4^. Locating the mandibular canal is not only important in the diagnosis of vascular and neurogenic diseases associated with the nerve^5^, but also in the diagnosis of lesions adjacent to the mandibular canal, and planning of oral and maxillofacial surgeries.

Therefore, preoperative treatment planning and simulation are necessary to prevent nerve injury. This can be achieved by identifying the exact location of the mandibular canal, that contains the IAN surrounded by thin cortical bone^5,6^.

Cone beam computed tomography (CBCT) is the most commonly used three-dimensional (3D) imaging modality for preoperative treatment planning and postoperative evaluation in dentistry^7^. The CBCT volume is reconstructed using projection images obtained from different angles with a cone-shaped beam and stored as a series of axial images^8^. CBCT can be used for observing and positioning anatomical structures with lower doses of radiation and lower costs, when compared to multi-detector computed tomography (MDCT)^9^. However, in practice, there are certain challenges associated with mandibular canal segmentation from CBCT images, such as inaccurate density and large amount of noise^10^.

Recently, deep learning has been utilized to precisely classify lesions and segment medical or dental images^11^. Furthermore, performance can be enhanced, surmounting the limitations of datasets, varied images, low resolution, etc., by using pre-trained models with multi-stream (multi-angle, multi-scale, multi-modality) and 3D image learning. In particular, it is considered that transfer learning enables training without overfitting on small target datasets to boost generalization, and initializing with transferred features is considered a useful technique to improve deep neural network performance^12,13^. Though processing 3D medical scans is a computational burden, multi-stream learning and 3D convolutional neural network (CNN) has also been widely used by researchers, in accordance with clinicians’ standard practice of rotating, zooming in/out of 3D images and checking adjacent images during diagnosis^14^. Although low-dose CT (LDCT) is compromised by lower image quality and diagnostic performance similar to CBCT, this method is widely used, owing to the lower X-ray dose than that in normal-dose CT (NDCT). Recently, researchers have successfully performed segmentation of LDCT by denoising images using 3D CNN^15^.

In the field of dentistry, a method to segment mandibular canal in panoramic radiography using deep learning was explored, which reported highly accurate results (0.847)^16^. This high accuracy reflects the benefits of learning 2D images, as the canal occupies a large portion of the overall image in 2D panorama and the resolution of panoramas is higher when compared to that of CBCT. However, the panorama has a limitation that it is challenging to reveal the actual three-dimensional rendering of a complex canal structure as the panorama highlights the canal from just one point of view. Therefore, detection and segmentation of the mandibular canal on CBCT images using various deep learning networks were attempted in this study to investigate the possibility of its clinical application. 2D^17^ and 3D U-Nets^18^, and 2D SegNet^14,19^, that are commonly used in the medical field to segment anatomical structures or pathological lesions, were utilized to segment the mandibular canal and analyzed from the perspective of time complexity and performance.

## Materials and method

### Ethics statement

This study was approved by the Institutional Review Board (IRB) of the University Dental Hospital (Approval number: PNUDH-2019-009). The IRB of the University Dental Hospital waived the need for individual informed consent, and thus, a written/verbal informed consent was not obtained from any participant, as this study had a non-interventional retrospective design and all the data were analyzed anonymously.

### Materials

In this study, images of 102 patients (aged 18–90 years) undergoing CBCT for TMJ diagnosis between 2008 and 2017 at the University Hospital were used. CBCT scans were performed using a PaX-Zenith 3D system (VATECH Co., Hwaseong, Korea) with 5.0–5.7 mA, 105 kV, a 24-s exposure time, a voxel size of 0.2–0.3 mm, and a field of view of 16 × 16 or 24 × 24. Patients presenting previous surgical history, malformation, or diseases of the oral and maxillofacial region were excluded.

Two trained researchers traced the mandibular canal in cross-sectional images using INVIVO^TM^ (Anatomage, San Jose, CA, USA) dental imaging software to generate a ground truth image. For the practical annotation processing, the INVIVO’s cross-sectional view was annotated at 1 mm intervals, following the restoration of the original interval of 0.2 mm using 3D cubic interpolation. An oral and maxillofacial radiologist, with 6 years of experience, clarified the positions of any uncertain mandibular canals. The original image was stored with a tracing image. The tracing image was then replaced with the ground truth label that consisted of the mask for the mandibular canal (white) and background (black). The canal mask was extracted using the color information from the tracing image.

### Methods

#### Preprocess

**Figure 1:**
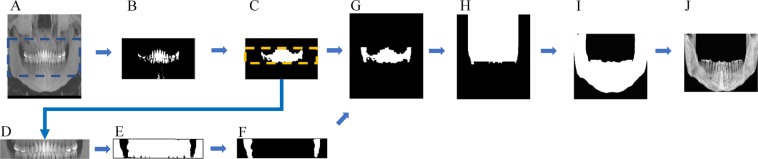
Preprocessing steps such that just the mandibular part remained.

The size of all dimensions was resized by half before the preprocess. In order to increase accuracy and at the same time reduce the volume that the network learns, preprocessing was performed such that automatically only the 3D mandibular part from the raw data remained (Fig. 1). First, the center one-third of the reconstructed panoramic view (2D) (A) was binarized with teeth threshold (B) and then dilation was performed, leaving only the largest object (C). The resulting image, confined to the tooth height (D), was binarized with bone threshold (E). Leaving the two largest images after complementing the image (F), a buccal corridor between the ramus and jaw bone was obtained. Next, this buccal corridor and the tooth part (C) was combined (G), the maxillary part was obtained by extending the image (H) upwards. The maxillary region was removed from the 3D binarized jaw bone image, and a 3D closing operation was performed to obtain a binarized mandibular image (I). After confining the area within the bounding box of this image, the mandible image was finally obtained, thus leaving only the inner part of the mask in the original image (J). In (A) and (D), the binarization coefficients of the teeth and bone were accurately calculated by limiting the area, and the binarization coefficients were obtained using the multi-level Otsu’s method. The bone threshold used the first level and the teeth used the third level threshold. In the case of inaccurate mandibular segmentation result, the threshold of the bone and tooth were manually readjusted.

### Networks

#### 2D Networks

**Figure 2:**
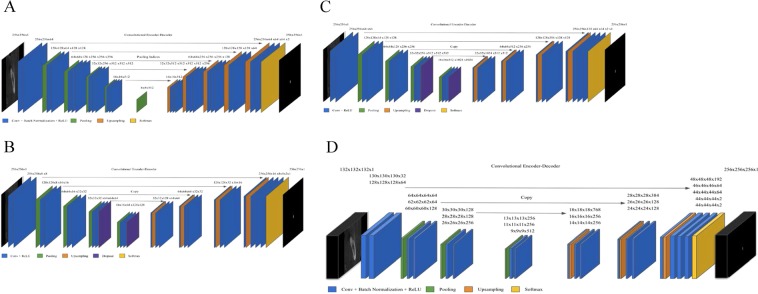
Architecture of deep learning networks. (**A**) SegNet; (**B**) U-Net with fewer filters than the original U-Net (**C**) U-Net with the original number of filters; (**D**) 3D U-Net.

First, two image segmentation architectures, U-Net and SegNet, that share similar encoder and decoder network architectures, except for some differences, were implemented. SegNet uses the basic architecture from VGGNet^20^ with the pre-trained convolutional layer and batch normalization, while its decoder uses the max pooling indices to up-sample the feature map instead of learning like Fully Convolutional Network (FCN)^21^. With dental images, the same number of filters were used as illustrated in Fig. 2A. In contrast, U-Net had up-sampling operators by learning to deconvolute the input feature map and combine it with the corresponding encoder feature map for a high-resolution feature map as the decoder output.

In this study, the original architecture of 2D U-Net was modified as follows: first, the feature maps were padded with zeros using the ‘same padding’ instead of the ‘valid padding’ in the convolutional layers, so that an input image was fully covered by specified filter and stride; next, the cropping process was removed before copying the drop-out or convolutional layer outcomes as shown in Fig. 2B,C. Even though the valid sampling and cropping processes were key points originally used in the U-Net algorithm to find the hidden pattern and convey to the deep network, in this study, the number of pixels of the desired detection area (mandibular canal) was very small and was located partially on the edge. Hence, padding feature maps filled with zeros were applied to maintain the same dimensions, to avoid over-fitting via class imbalance and information loss of the edge surrounding the mandibular canal at the corner^22^. The 2D U-Net was first studied with both small and original number of filters as the original U-Net and SegNet. The 2D U-Net architecture with original filters was then experimented with pre-trained weights from 2D VGG net. Additionally, 2D U-Net pre-trained from 2D VGG net was examined with 4 adjacent images. Given the fact that doctors see adjacent images when diagnosing 3D images, this network structure was expected to obtain more contextual information from 2D adjacent images and simultaneously circumvent the computation burden of 3D training.

#### 3D network

A 3D U-Net^18^ fully convolutional network for 3D canal segmentation was also used. It is an extension of the 2D U-Net layers in 3D (Fig. 2D), which was learned by randomly selecting 64 3D patches with each image of the size 132 × 132 × 132 pixels. The 3D U-Net used the same 2D U-Net architecture, with corresponding 3D operations (3D convolutions, 3D max pooling, and 3D up-convolutional layers)^18^, batch normalization addition, and dropout layer removal. Since 3D network gets more contextual information, it maintains valid padding as its original 3D architecture.

### Training options

With binary cross entropy, class weight of 5.3:1000 was used in all networks to compensate class imbalance by using the pixel label counts. We used median frequency balancing^23^ for calculating the class weight as proposed in SegNet. Of the 49094 images, the dataset was divided into train:valid:test sets with the ratio of 6:2:2, and train:valid:test datasets had equal class images. Each image originally had 545 × 900 pixels, and it was used as 256 × 256 pixels for 2D and 132 × 132 × 132 pixels for 3D. NVIDIA Titan RTX GPU with cuDNN version 5.1 acceleration was used for 3D network training.

#### 2D network

The U-Net and SegNet were trained with and without a pre-training class weight individually. The U-Net was first studied with 1) fewer filters than retaining the original U-Net architecture and 2) larger number of filters (deeper network) of the original U-Net and SegNet. Moreover, with the original SegNet architecture, pre-trained weights from VGG net could be used not only for SegNet but also for U-Net. Therefore, U-Net and SegNet with 1 image, U-Net with 4 adjacent images were studied with transfer learning. The models were trained for 600 epochs with Adam optimizer^24^ with a learning rate and decay (0.01, 0.005) for SegNet and (0.0001, 5e^−4^) and a momentum of 0.9 for U-Net instead of 0.99 from U-Net’s original proposed momentum. We trained the 2D variants until the training loss converged. The model with the best performance on a validation dataset was selected.

#### 3D network

The models were trained for 100 epochs using Adam optimizer^24^ with a learning rate of 5e^−4^ decayed by a factor of 5 after 5 epochs, and a batch size of 8.

### Metrics for accuracy comparison

The canal and background pixel accuracy, global accuracy, class accuracy, and mean IoU (intersection over union) were assessed to evaluate the accuracy. Each definition is as follows:

$${\rm{Pixel}}\,{\rm{accuracy}}\,{\rm{of}}\,{\rm{canal}}=\frac{{\rm{TP}}}{{\rm{TP}}+{\rm{FP}}}$$

$${\rm{Global}}\,{\rm{accuracy}}=\frac{{\rm{TP}}+{\rm{TN}}}{{\rm{TP}}+{\rm{TN}}+{\rm{FP}}+{\rm{FN}}}$$

$${\rm{Class}}\,{\rm{accuracy}}={\rm{average}}\,{\rm{of}}\,{\rm{pixel}}\,{\rm{accuracy}}\,{\rm{of}}\,{\rm{canal}}\,{\rm{and}}\,{\rm{background}}$$

$${\rm{IoU}}\,{\rm{of}}\,{\rm{canal}}=\frac{{\rm{TP}}}{{\rm{FN}}+{\rm{TP}}+{\rm{TN}}}$$

$${\rm{Mean}}\,{\rm{IoU}}={\rm{average}}\,{\rm{of}}\,{\rm{IoU}}\,{\rm{of}}\,{\rm{canal}}\,{\rm{and}}\,{\rm{background}}$$

$${\rm{TP}}:{\rm{true}}\,{\rm{positive}},{\rm{FP}}:{\rm{false}}\,{\rm{positive}},{\rm{FN}}:{\rm{false}}\,{\rm{negative}},{\rm{TN}}:{\rm{true}}\,{\rm{negative}}$$

## Results

The quantitative results in Table 1 show the importance of class balancing, pre-training, and the performance of each network. The final pre-trained 2D U-Net with the original number of filters achieved a global accuracy of 0.84 and 0.82 and class average accuracy of 0.63 and 0.68 for each network using 1 image and 4 adjacent image cases. The SegNet was also tested with and without pre-trained layers with class weights, and the pre-trained SegNet model showed the highest accuracy of 0.96 and class accuracy of 0.90 among 2D networks. 3D U-Net showed the best results on all accuracy indexes including global accuracy (0.99) and class accuracy (0.96).

**Table 1:** The performance comparison of test results using background (BG), mandibular canal (MC), global accuracy, class average accuracy, and mean of intersection over union (mIoU).

	BG	MC	Global acc	Class acc	mIoU
2D SegNet*^,^^	**0.96265**	**0.84278**	**0.96254**	**0.90271**	**0.49116**
2D U-Net	0.76764	0.50470	0.76741	0.63617	0.38462
2D U-Net*	0.91744	0.26388	0.91686	0.59066	0.45984
2D U-Net*^,^^	0.83897	0.42008	0.83859	0.62953	0.42043
2D U-Net (adjacent 2 images)*^,^^	0.82013	0.54608	0.81988	0.68310	0.41125
3D U-Net	**0.99972**	**0.92738**	**0.99922**	**0.95915**	**0.57721**

^*^The same number of filters (same as the original SegNet and UNet).

^Pre-trained weights with natural images.

**Figure 3:**
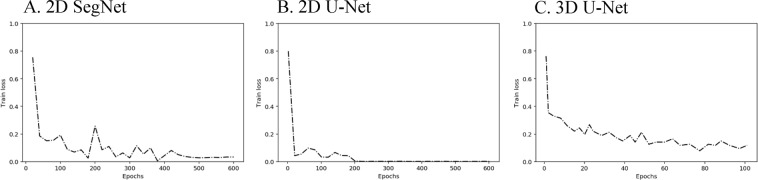
Training progress of each network. (**A**) 2D SegNet; (**B**) 2D U-Net; (**C**) 3D U-Net Each model was stopped when its training loss converged (**A**) Training loss of pre-trained SegNet with 600 epochs; (**B**) Training loss of non-pre-trained SegNet with 600 epochs; (**C**) Training loss of U-Net with 600 epochs. (**D**) Training loss of 3D U-Net with 100 epochs.

As seen in Fig. 3, there was a fast convergence of global loss of the training data set in all the charts within 600 epochs. Particularly, as the authors highlighted the speed of model, 2D and 3D U-Nets used graphic processing unit (GPU) memory more than SegNet, but provided a quick model with the 0.2 level-loss in 20 epochs. With respect to performance, SegNet with pre-training approached the 0.2 error rate in 30 epochs, and the models gradually converged to the 0.1 level of error rate.

**Figure 4:**
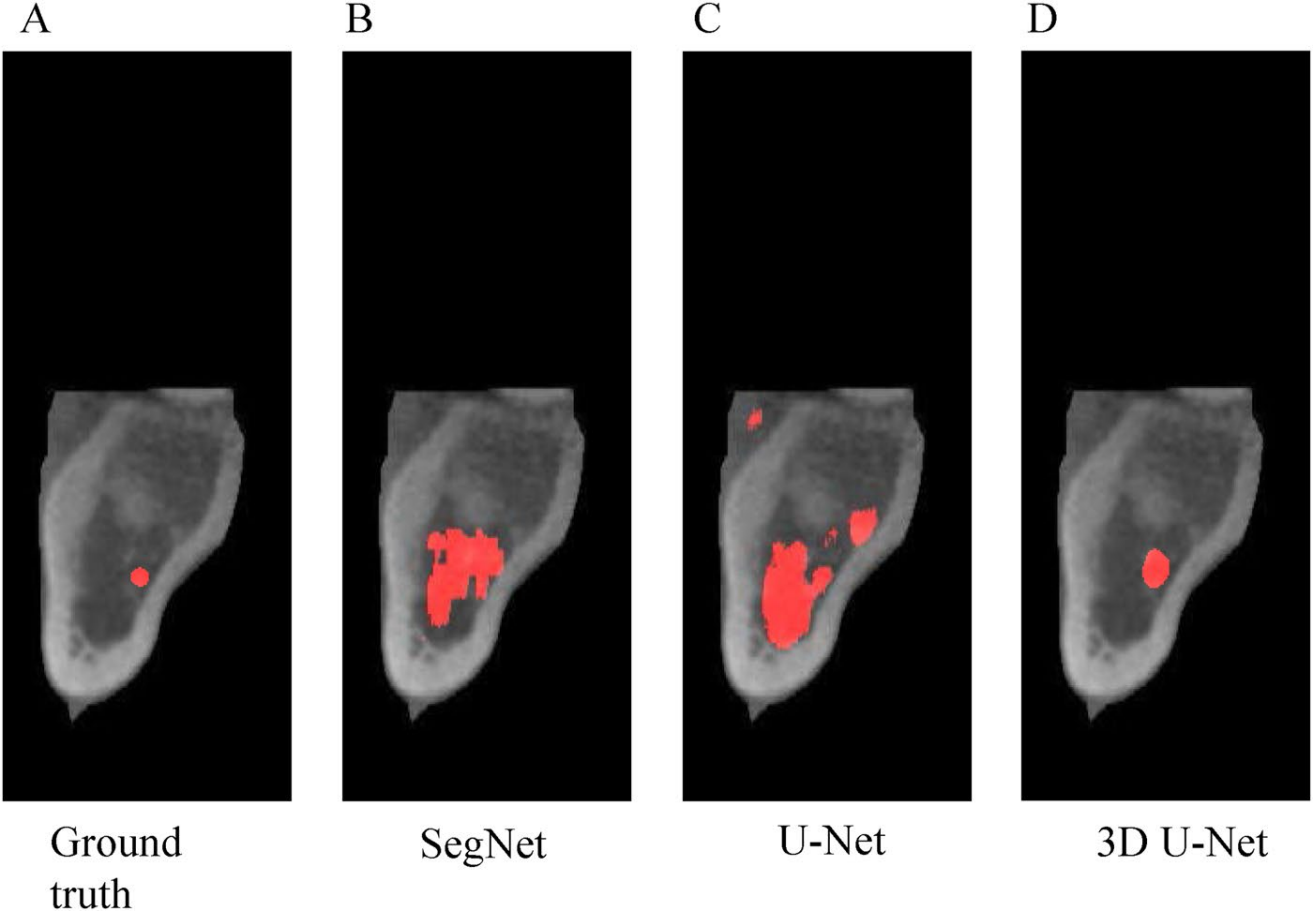
Segmentation result in the slice containing 2^nd^ molar. From left to right, test input image (**A**) ground truth mask; (**B**) 2D SegNet segmentation result; (**C**) 2D U-Net segmentation result; (**D**) 3D U-Net segmentation result.

**Figure 5:**
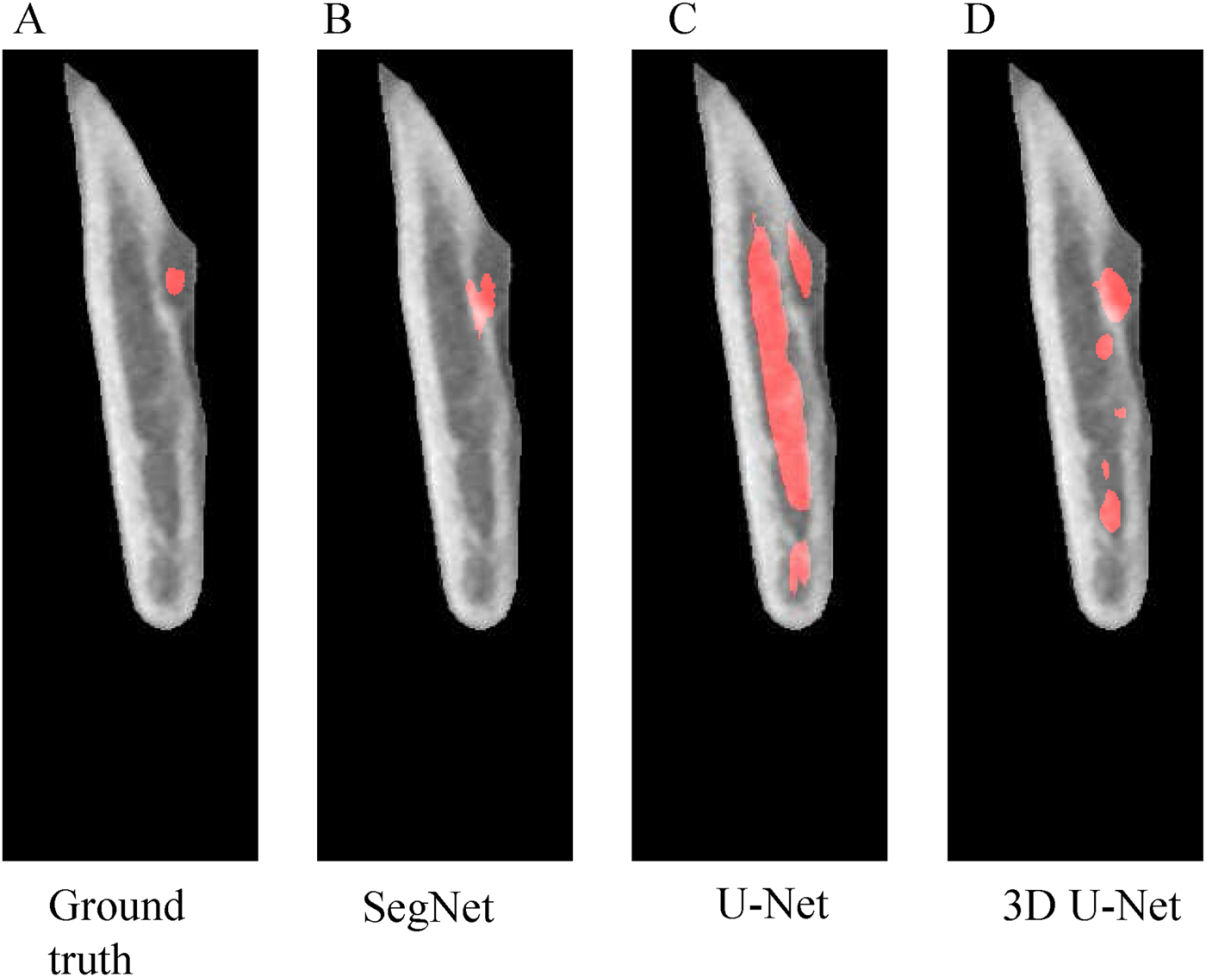
Segmentation result in the slice containing mandibular foramen area. From left to right, test input image (**A**) ground truth mask; (**B**) 2D SegNet segmentation result; (**C**) 2D U-Net segmentation result; (**D**) 3D U-Net segmentation result.

After the validation set confirmed each model’s convergence, all 9818 test images were used for testing and the results are shown in Figs. 4 and 5. The ground truth mask for the mandibular canal was small (Figs. 4A, 5A), and the SegNet sensed the area and segmented it more accurately than 2D U-Net. However, in contrast to the high-resolution purpose of the up-sampling section, the prediction area of 2D U-Net was heavily emphasized and its performance was notably lower than SegNet, especially when the cortical layers beside the canal is thicker and more clear (Figs. 4B,C, 5B,C). Though 3D U-Net demonstrated the best performance, it was not able to detect the canal when the surrounding cortical layer was ambiguous (Figs. 4D, 5D).

## Discussion

While detection of the IAN is a crucial step in dentistry for implant treatment planning and any other oral and maxillofacial surgery, it is usually identified by manual positioning in each cross-section of CBCT images. Although CBCT is widely used to detect the mandibular canal and analyze the relationship with other important structures, it has the following limitations:

1. Unlike CT, though CBCT has high resolution, it does not measure the density accurately (Hounsfield unit) and is more susceptible to noise^10^. Furthermore, the contrast is lower than that of CT, which makes detecting anatomical structures^25^ automatically, comparatively harder.
2. Since the mandibular canal travels in various directions in three dimensions^26,27^, it is difficult to know the exact shape of the canal in a single direction. The challenging part is the change in the location and shape of the mandibular canal in cross-section images due to changes in the head position.
3. If the cortical layer around the mandibular canal is thin or the medulla pattern is not clear, it may be difficult to distinguish the canal shape^28^.
4. It is difficult to clearly distinguish from the root of teeth, when in contact or overlapping with the adjacent teeth such as 3rd molar^29^.
5. It is a time-consuming task to manually annotate the canal mask from each transverse slice at the pixel level.

Despite these difficulties, various algorithms have been proposed, that can be categorized into two methods: statistical shape methods (SSM) and atlas-based segmentation (ARS) methods^30^. SSM uses parametric variable shape models for canal segmentation^31^, while ARS deforms the atlas image into object images with non-rigid registration and segments the canal area from the scanned image^32^. The principal difference between ARS and SSM is that the former is independent of prior knowledge, whereas the latter achieves segmentation by exploring prior knowledge such as shape information. The limitation of ARS is that it may fail to handle new forms of data beyond the atlas. In particular, ARS registers and segments the mandible slice by slice, that partly solves the problem of the non-standard position. In contrast, SSM utilizes some prior knowledge to reconstruct a 3D model based on CBCT images, and its performance largely depends on the 3D reconstruction method that is adopted. Both SSM and ARS depend on either prior knowledge or other preprocessing techniques, and thus may fail in handling new incoming data that do not satisfy the predefined assumptions.

To increase accuracy to the extent where clinical application is feasible, it is preferable to adopt a new method that is fully data-driven and conducts the end-to-end segmentation. Deep learning satisfies the aforementioned conditions, and in recent years, it has demonstrated high accuracy in medical image recognition and segmentation^33^. Therefore, in this study the mandibular canal was segmented automatically using 2D and 3D deep learning networks. With reference to intervention from the authors, the background class dominated majority of the pixels in an image, and the mandibular canal occupied a very small part of the image and appeared infrequently. However, by applying a class weight, preprocessed (cropped) datasets, and batch normalization, the authors tried to overcome the class imbalance issue in this paper.

The results from 2D network suggest that deep learning can be applied to the segmentation of a small object such as the mandibular canal. The results also demonstrated that pre-training and optimization of a class weight, in conjunction with each network’s unique characteristics, can improve segmentation accuracy. Specifically, SegNet with a class weight of 5.3:1000 with pre-training showed the highest global and class accuracy among 2D networks, which can be useful to a certain extent at detecting fine segments. Unlike SegNet that re-uses pooling indices for up-sampling, batch normalization, and transfer learning, the predicted mask of U-Net has a noted tendency to converge to zero with a high contribution of segmentation towards the background. In worst cases, the middle kernels in the network merely leave black or big white region all over the jaw bones. Pooling indices and batch normalization of SegNet helped overcome this overfitting problem to some extent. Although U-Net was suggested by the medical image community to overcome the lack of annotated images with medical image specialized architecture, the results of this study suggest that 2D U-Net is not a desirable choice when detecting small 3D anatomical structures surrounded by thin or unclear cortical layer.

It is generally expected for 3D CNN to generate more accurate results as it is able to learn contextual information between image slices of complex 3D anatomical structures^18^. However, there needs to be two questions answered for 3D network to be clinically applied to mandibular canal segmentation. The first question is with reference to the computing power and time needed for training 3D CNN, sometimes 8 to 32 times^34^ as long. The GPU memory requirement is especially high for 3D medical images of 512 × 512 × 512 pixels. The authors tried to overcome this obstacle by confining the training volume to a mandibular part and successively reducing it to almost one-third of the original volume. The second question is with reference to the usefulness of the 3D network when applied to very small structures while we trade-off computation power and complexity. As there has been no obvious evidence of the performance gain of 3D CNN over its 2D counterpart for very small and complex structures such as the mandibular canal, three step experiments were conducted to evaluate the performance: the original 2D network, 2D network with adjacent images, and 3D network. 2D naïve U-Nets in this study showed comparatively high global accuracy but recorded lower class and mean IoU primarily because they are compelled to ignore the spatial contexts in the third dimension^18^. Using transferred features from natural images results in better output than naïve 2D architecture. The information from adjacent images with sharing weights also helped to learn the task, which exhibits promising results for future research. 3D U-Net recorded the highest accuracy in every index (Table 1) and about 18% higher mean IoU than the best 2D model of SegNet considered in this study.

^14^When comparing the high and low IoU area of the deep learning network (Figs. 4 and 5), it was found that the IoU was low where the cortical layer around the canal was not clear to the naked eye. As the cortical layer itself is a key feature, the network performance was poor in instances when the cortical structure was too thin or ambiguous. Even after conveying the path of U-Net for capturing high resolution objects, unclear parts of cortical layers’ edges surrounding the canal affected the performance of information transfer from encoder to decoder. 3D networks also exhibited limitations when the cortical layer around the canal was not clear (Fig. 5). In such cases, the 3D network soon lost the information about the canal and focused on other distinct cortical area during convolutional and pooling layer.

Though 3D U-Net showed significantly better results than 2D Networks, the GPU memory requirement for 3D CNN after volume reduction is still considerably high for local hospital and clinical environments. 2D U-Nets and SegNet demonstrated the potential of 2D networks for higher accuracy by combining improved network architecture and semi-contextual information. Future research on further improving efficiency is expected using projected 2D images such as reformatted panoramic views. These projected 2D images can be a key to reducing the computation burden using compressed information.

Once the mandibular canal is detected automatically and accurately by the dental imaging software through deep learning, which will be further explored in a future study, the authors expect that it will be significantly useful in everyday clinical diagnosis and treatment planning. This study could be viewed as a preliminary report to encourage a new opportunity to apply deep learning in segmentation of small and complex structures using CBCT images.

## Data Availability

The data that support the findings of this study are available on request from the corresponding author, JJH. The data are not publicly available because they contain information that could compromise the privacy of research participants.
